# High-Emissivity Double-Layer ZrB_2_-Modified Coating on Flexible Aluminum Silicate Fiber Fabric with Enhanced Oxidation Resistance and Tensile Strength

**DOI:** 10.3390/ma17133234

**Published:** 2024-07-01

**Authors:** Wei Li, Xueying Zhang, Liwen Yan, Anran Guo, Haiyan Du, Jiachen Liu

**Affiliations:** Key Laboratory of Advanced Ceramics and Machining Technology of Ministry of Education, School of Materials Science and Engineering, Tianjin University, Tianjin 300072, China; liwei3411@tju.edu.cn (W.L.);

**Keywords:** double-layer coating, ZrB_2_, thermal barrier coating, composite fiber felt, mechanical strength

## Abstract

Fibers crystallize and become brittle at high temperatures for a long time, so the surface coating must maintain long-lasting emission performance, which requires superior antioxidant properties of the high-emissivity fillers. To improve the radiation performance of the coating and the tensile strength of the fiber fabric, a double-layer coating with high emissivity was prepared on the surface of flexible aluminum silicate fiber fabric (ASFF) using MoSi_2_ and SiC as emissive agents. The incorporation of borosilicate glass into the outer coating during high-temperature oxidation of ZrB_2_ results in superior encapsulation of emitter particles, effectively filling the pores of the coating and significantly reducing the oxidation rate of MoSi_2_ and SiC. Furthermore, the addition of an intermediate ZrO_2_ layer enhances the fiber bundle’s toughness. The obtained double-coated ASFF exhibits an exceptionally high tensile strength of 57.6 MPa and a high bond strength of 156.2 kPa. After being subjected to a 3 h heating process, the emissivity exhibits a minimal decrease of only 0.032, while still maintaining a high value above 0.9. The thermal insulation composites, consisting of a flexible ASFF matrix and a ZrB_2_-modified double-layer coating, exhibit significant potential for broad applications in the field of thermal protection.

## 1. Introduction

During near-space flight and re-entry, the compression of the high-speed airflow and high-speed dynamic friction between the hypersonic aircraft and the atmosphere will generate high thermal energy, namely aerodynamic heating [1,2,3,4,5]. Consequently, effective thermal protection is imperative to mitigate inward heat transfer, such as using carbon fiber composite materials and high-temperature-resistant alloy materials, designing cooling systems, or using insulation layers to ensure the safe operation of an aircraft’s internal components [6,7,8]. The thermal protection system (TPS) on the leeward side of the spacecraft usually consists of a flexible felt fiber and a high-emissivity coating that dissipates and isolates heat energy. The felt fiber demonstrates a layered quilted structure comprising two layers of fiber cloth enveloping an internal layer of cotton fiber, which are meticulously stitched together using fiber threads [9,10]. The felt fiber exhibits low thermal conductivity and marvelous flexibility, enabling it to tightly adhere to the fuselage surface. Nonetheless, under prolonged use at high temperature, the fiber undergoes crystallization, inducing functional failure detrimental to application stability. A coating spayed on the surface can effectively improve the temperature resistance of the fiber blanket by radiating the heat, which is important in ensuring the reusability of TPS [11,12].

The key constituents of a high-emissivity coating encompass a filler with high emissivity and the binder. The emissivity depends on the temperature and type of the material, as well as its surface condition, with the material itself being the most direct factor determining the emissivity. MoSi_2_, TaSi_2_, SiC, SiB_4_, and SiB_6_ are potential options for high-emitter fillers [13,14,15,16,17]. Guo et al. prepared silica-coated MoSi_2_ powder through a sol–gel process. The theoretical thickness of the cladding shell layer was about 60 nm, and the MoSi_2_@SiO_2_ coating showed satisfactory isothermal cyclic oxidation resistance at 400–600 °C for 12 h, with an oxidation weight gain rate of less than 1 wt.% [18]. Gao et al. mixed SiB_6_ and Si into TaSi_2_ and SiC powders for modification and then prepared a high-emissivity coating with high antioxidant properties on alumina fiber fabric (AFF). The borosilicate glass phase generated by SiB_6_ oxidation at high temperatures healed the cracks on the coating and encapsulated the emitter. After calcination at 1200 °C, the tensile strength of the AFF with 2.5% SiB_6_ increased by 85%, while the average spectral emissivity in the 2–14 μm band reached 0.95 at room temperature (RT) [19]. Shao et al. prepared a multiphase coating of MoSi_2_–ZrO_2_–borosilicate glass with SiB_6_ by slurry impregnation and sintering, and the resulting coating formed a gradient structure. The dense surface layer provided oxidation resistance and a waterproofing effect, while the internal porous layer greatly enhanced the thermal shock resistance and the adhesion of the coating to the substrate. The coating with 3 wt.% SiB_6_ added had an emissivity of 0.85 in the 0.8–2.5 μm band at RT [20]. Zhang et al. used SiC and ZrO_2_ as emissive agents to prepare a bilayer coating with high emissivity on the ASFF. Compared with the original fabric, the double-layer coated fabric had 70% higher tensile strength (75 MPa) after calcination at 1100 °C. Phase analysis after heat treatment revealed that the thermal resistance of fiber fabric markedly increased, with the emissivity in the short-wavelength range reaching 0.93 [21]. These studies consider antioxidant measures when using silicide emitters, but in cases where the fiber clusters on the fiber cloth are bent and uneven, it is necessary to consider the density and continuity of the coating shell at high temperatures.

MoSi_2_ and SiC are emitters commonly used for high-emissivity coatings but are ball-milled to fine powders before being added to the coating. The high specific surface area and reactivity of the resulting powders are therefore susceptible to oxidation [22,23]. To reduce the exposure of MoSi_2_ and SiC to oxygen, a cladding structure is usually formed by in situ generation or viscous flow phase coverage [24,25]. ZrB_2_ has been extensively studied in ultra-high-temperature ceramics, especially ZrB_2_–SiC composites, and the synergistic effect of oxidization revealed a complex process involving the vapor-based transport of SiO and B_2_O_3_ gas, liquid B_2_O_3_ transport, and the precipitation of dissolved ZrO_2_ [26,27]. The glassy oxide formed a protective oxide skin, so the incorporation of ZrB_2_ into MoSi_2_ and SiC powders may be an effective way to reduce the oxidation rate [28]. Nowadays, coating preparation methods usually utilize multiple emissivity agents, as the synergistic and supplementary effects of the radiation characteristics in the high-emissivity material composite at different temperatures and bands can enhance the overall radiation performance of the composite coating [3,29,30,31].

An overly dense coating is not conducive to the flexibility of fibers, but a less dense coating can easily exacerbate the oxidation of the emitter, so a porous intermediate layer is added in this work [32]. ZrO_2_ is an oxide with high emissivity in the 8–14 μm band, a high melting point, and low thermal conductivity [33,34]. Monocyclic ZrO_2_ (m-ZrO_2_) converts into tetragonal ZrO_2_ (t-ZrO_2_) at temperatures above 900 °C. These metastable t-ZrO_2_ particles can exist stably in the coating and undergo martensitic transformation under pressure to absorb energy and improve the toughness of the material [35,36]. ZrO_2_ can similarly serve as a network-forming agent and transform the peak of [BO_3_] to [BO_4_] in borosilicate glass, improving the network structure and chemical stability of the glass [37,38,39].

Rigid glass is widely employed as a bonding agent for high-temperature insulation coatings due to its excellent thermal stability, mechanical properties, and self-healing ability [40]. However, using glass powder as a binder raw material necessitates high-temperature sintering, which is prone to matrix shrinkage deformation and cristobalite phase precipitation from the glass during cooling [29,41]. Comparatively, the utilization of silica sol as a binder material significantly reduces the required curing temperature while maintaining reliable bonding performance at elevated temperatures. Therefore, it is better suited for collaboration in the context of flexible felt fiber with a relatively limited temperature resistance [42,43].

In this work, two emitters, MoSi_2_ and SiC, are selected to promote the broad-spectrum radiation performance of the coating. Furthermore, the neutral silica sol as a binder lessens the density of the coating due to the shrinkage of the gel during curing, which leads to cracking. Therefore, ZrB_2_ is added to form a borosilicate glass phase at elevated temperatures to improve the density and oxidation resistance of the coating. At the same time, the ZrO_2_ silica sol coating was prepared as an intermediate layer to mitigate glass-to-fiber bonding failure during melting. The effects of coatings containing varying ZrB_2_ content on the tensile strength, bonding strength between the coatings and fabric, and thermal performance of the ASFF after heat treatment at different temperatures were investigated. The cross-sectional morphology of the double-layer coating was analyzed following high-temperature calcination, while the tensile strength and radiation properties of the single-layer and double-layer fabric coatings were compared.

## 2. Materials and Methods

### 2.1. Materials

Aluminum silicate fiber fabric (ASFF; 0.26 ± 0.02 mm thickness, warp and weft density of the plain fabric, 20 × 20 roots/cm^2^; Hubei Feilihua Quartz Glass Co., Ltd., Jingzhou, China) was used as the matrix for the series of experiments in this study. MoSi_2_ (3–5 μm, 99%), SiC (3–5 μm, 99%), ZrB_2_ (3–5 μm, 99.5%), and ZrO_2_ (0.5 μm, 99.9%) powders were provided by Eno Materials (Cangzhou, China). Octanol and polyacrylamide were provided by Tianjin Xiensi Biochemical Technology Co., Ltd. (Tianjin, China), which were used as defoamer and dispersant agents, respectively. The solid content of neutral silica sol (Zhejiang Zhiti Nawei New Material Co., Ltd., Taizhou, China) was 25%, with a particle size of 10 nm and a pH of 7. All chemicals and raw materials were used as received. 

### 2.2. Preparation of the Double-Layer Coating

**Figure 1:**
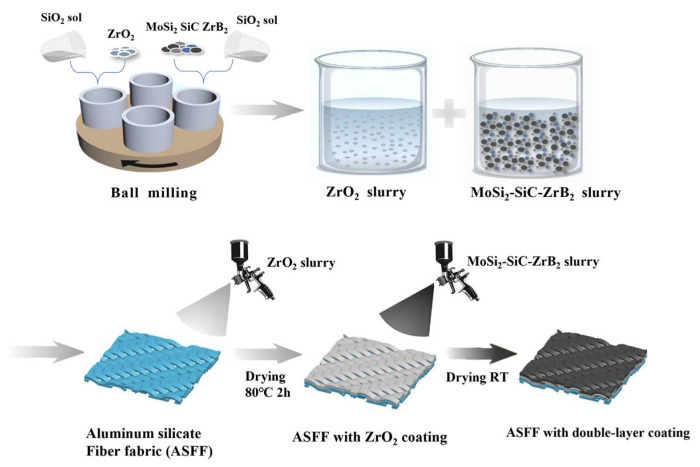
Flow chart of the preparation processes of the double layer on the ASFF surface.

The double-layer coatings were prepared as in Figure 1. Polyacrylamide (0.5 wt.%) and octanol (0.5 wt.%) were added to neutral silica sol as dispersing and defoaming agents, respectively, and the sol was mixed evenly by magnetic stirring. MoSi_2_, SiC, and ZrB_2_ powders were then mixed at different mass ratios (Table 1), added to the mixed silica sol, and stirred on a ball mill at 300 r/min to obtain a uniform and stable coating mixture. The coating paste was uniformly sprayed on the ASFF at a loading rate of 60–80 g/m^2^ and dried at RT for 8 h to obtain a single layer of MoSi_2_–SiC–ZrB_2_ coated fabric for subsequent study. In addition, a ZrO_2_ coating slurry was prepared by mixing ZrO_2_ powder and silica sol at a 1:1 mass ratio. The ZrO_2_ slurry as an intermediate layer was evenly sprayed on the surface of the ASFF with a load of 50–70 g/m^2^. After drying at 80 °C for 2 h, the MoSi_2_–SiC–ZrB_2_ coating with 5% ZrB_2_ was sprayed onto the middle layer to obtain a double-layer coating. 

### 2.3. Characterization and Testing

The phase composition of the coating surface was identified by X-ray diffraction (XRD, D/Max-2500, Rigaku, Akishima-shi, Japan) using Cu-Kα radiation in the range of 2θ = 10–60°. The phases from the XRD spectrum were identified by using the Powder Diffraction File (PDF) database of the International Diffraction Data Center (ICDD). Scanning electron microscopy (SEM) coupled with energy dispersive spectrometry (SEM/EDS, Hitachi SU1510, Tokyo, Japan) was used to survey the coating surface morphology and perform elemental analysis. Fourier-transform infrared spectroscopy (FTIR) was recorded on a NEXUS-670 Fourier transform infrared spectrophotometer using KBr pellets. The samples were prepared in 0.2–0.25 mm thick KBr pellets (1 mg in 100 mg of KBr). Based on the GB/T 1447-2005 standard [44], the tensile strength of the coated ASFF was tested using a mechanical testing machine (LD24.204, Lishi Instruments Co., Ltd., Shanghai, China) at a loading speed of 0.5 mm/min. Five specimens from each group were selected to test the mechanical properties, and the average value was calculated. The bonding strength between the coating and substrate was measured using an electronic universal testing machine. The substrate with dimensions of 30 × 30 mm was covered with the coating, and the fabric was adhered to an aluminum plate with 3M double-sided tape to conduct combined strength tests according to the GB/T 5210-2006 standard [45]. The infrared absorption spectra of the specimens were collected at RT on an infrared spectrometer (SR-5000 N, CI Systems, Jerusalem, Israel), with the spectral range of the thermal radiation emitted from the surface ranging from 2 to 14 μm. The thermal insulation properties of the flexible felt fiber were tested using a thermal infrared imager (LT7-P, Zhejiang Dali Technology Co., Ltd., Hangzhou, China).

## 3. Results and Discussion

### 3.1. SEM Images and XRD Spectra of MoSi_2_–SiC–ZrB_2_ Powders after High-Temperature Heat Treatment

**Figure 2:**
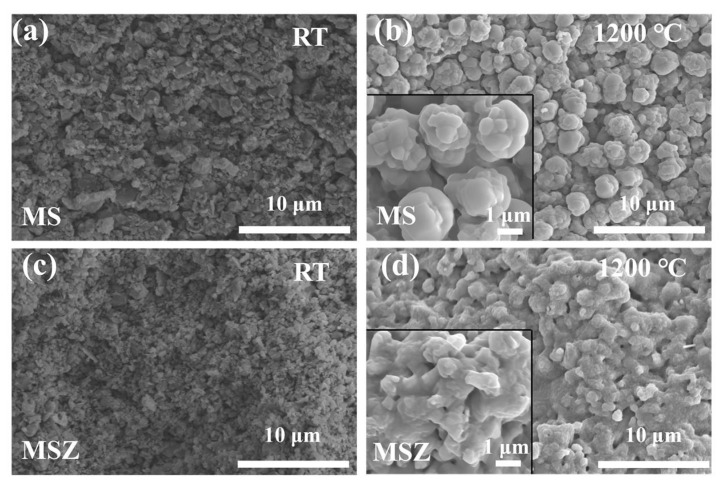
SEM images of the MoSi_2_–SiC (MS) and MoSi_2_–SiC–ZrB_2_ (MSZ) powders after heat treatment at 1200 °C: (**a**) MS-RT; (**b**) MS-1200 °C; (**c**) MSZ-RT; (**d**) MSZ-1200 °C.

To demonstrate that ZrB_2_ can generate B_2_O_3_ to shape the borosilicate glass phase with molten SiO_2_ in a high-temperature environment, two sets of MoSi_2_, SiC powder, and additional ZrB_2_ powder samples were ball-milled with water, dried, and heat-treated for 1 h at 1200 °C. These two different mixed powders were named MS and MSZ, respectively. The SEM images of the samples at RT and after heat treatment at 1200 °C are shown in Figure 2. There seems to be no significant discrepancy between the two types of particles at RT (Figure 2a,c). The emitter particles of the MoSi_2_–SiC sample undergo oxidation in large quantities after 1200 °C, and the generated square quartz phase aggregates on the particle surfaces to form larger independent microspheres (Figure 2b) [23]. In comparison, the MSZ sample with added ZrB_2_ after heat treatment has flowing parts wrapped around and connected with each particle. The main component of these flowing parts is the borosilicate glassy phase (Figure 2d).

**Figure 3:**
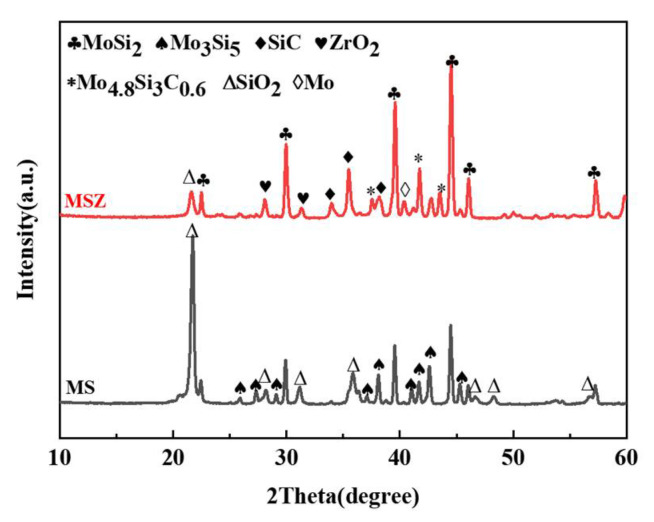
XRD patterns of the MS and MSZ powders after heat treatment for 1 h at 1200 °C.

The XRD spectra of the samples after heat treatment at 1200 °C are shown in Figure 3. The intensities of the characteristic MoSi_2_ and SiC peaks in the MS spectrum exhibit a significant reduction compared to those observed for the MSZ sample. The simultaneous emergence of prominent peaks corresponding to SiO_2_ and Mo_5_Si_3_ indicates the extensive oxidation of both emitters. The MSZ sample spectra exhibit novel peaks corresponding to elemental Mo and Mo_4.8_Si_3_C_0.6_, as the oxidation of ZrB_2_ removes most of the O_2_ and prevents the complete oxidation of MoSi_2_. Concurrently, the formation of a glassy phase comprising SiO_2_ and B_2_O_3_ occurs, effectively encapsulating the emitter particles and facilitating the combination of Mo_5_Si_3_ with SiC to yield Mo_4.8_Si_3_C_0.6_. The presence of this enclosed glass phase serves as a barrier against further oxidation of the emitter. In addition, the Mo_4.8_Si_3_C_0.6_ compound exhibits a high melting point of 2100 °C and demonstrates excellent chemical compatibility with SiC and MoSi_2_ at elevated temperatures, enabling it to effectively impede the passage of O_2_ [46,47].

### 3.2. Morphology and XRD Spectra of Single-Layer MoSi_2_–SiC–ZrB_2_ Coating and Double-Layer Coatings after Heat Treatment at 1200 °C for Different Times

**Figure 4:**
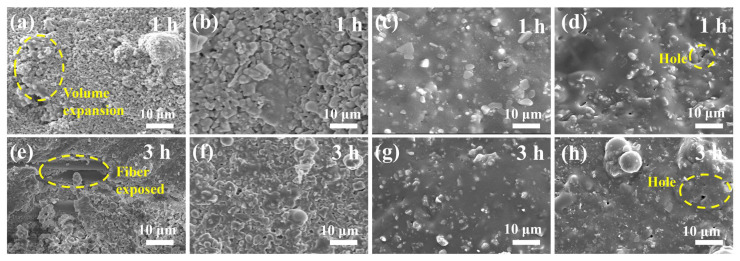
SEM images of the single-layer coating surface after heat treatment at 1200 °C for different times: (**a**,**e**) MSS-0Z; (**b**,**f**) MSS-3Z; (**c**,**g**) MSS-5Z; (**d**,**h**) MSS-7Z.

The surface morphologies of the single-layer coatings with different ZrB_2_ content after curing are similar, therefore they are not presented, and the surface micro-morphologies after heat treatment at 1200 °C for 1 and 3 h are shown in Figure 4. Under the same heating time, the coating surface containing more ZrB_2_ exhibits denser and fewer pores. The external layer of the MSS (MoSi_2_–SiC–Silica sol) coating exhibits a non-dense structure, characterized by particle stacking morphology with prominent pores and bulges resulting from the release of volatile by-products (MoO_3_ and CO_2_) and volume expansion-induced protrusions (Figure 4a). The further oxidation of MoSi_2_ and SiC, along with the contraction of silica sol, leads to the explosion of some fibers under the coating [48]. The exposed fibers are directly subjected to the high-temperature environment, rendering them susceptible to crystallization (Figure 4e). Alongside the emergence of ZrB_2_-induced flow phases, the glass phase generated at high temperatures promotes particle rearrangements, leading to a denser and smoother coating surface (Figure 4b,c). The borosilicate glass phases can envelop the emitter, preventing oxidation and sealing cracks that may arise during coating gel contraction, thereby reducing or inhibiting heat flow to the interior. The surface morphologies of MSS-3Z coating samples burnt for 1 and 3 h were compared. It was observed that there were additional cristobalite phases because the particles were no longer obvious but were encapsulated and connected by the flowing phase. Furthermore, the silicon–boron ratio in the bonding phase gradually increased, leading to an increase in the softening temperature with prolonged heating duration (Figure 4b,f). The presence of these softened glass phases facilitated the densification process of the coating. After being held at 1200 °C for 1 h, holes reappeared on the surface of the MSS-7Z coating (Figure 4d). Excess B_2_O_3_ resulting from the oxidation of ZrB_2_ was readily volatilized, leading to the formation of residual small pores that serve as channels for oxygen diffusion, while internal fillers’ oxidation caused a loss of flatness in the external coating.

**Figure 5:**
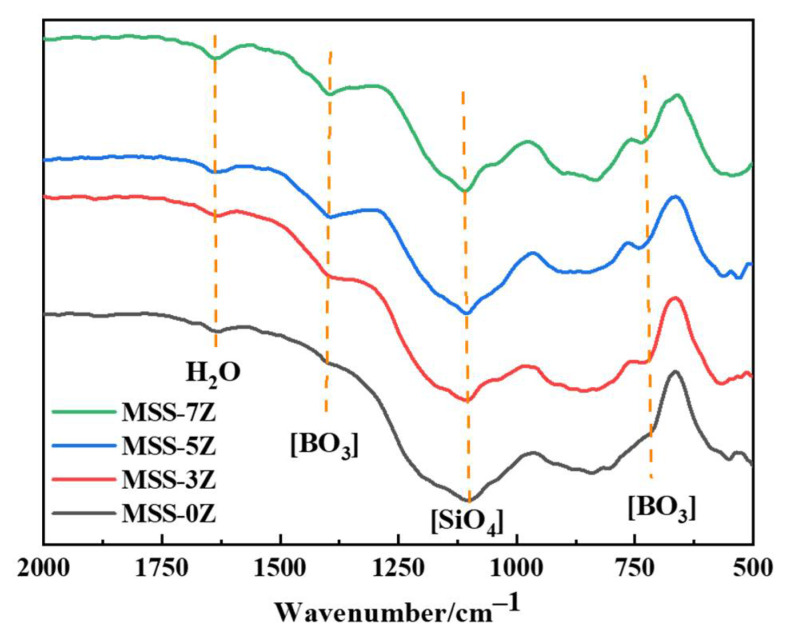
FTIR spectra of single-layer coatings with different ZrB_2_ content after calcination at 1200 °C for 1 h.

Figure 5 shows the infrared spectra of single-layer coatings with different ZrB_2_ contents after 1 h of heat treatment at 1200 °C. The orange dashed line in the figure corresponds to the chemical bond associated with the characteristic peak of this wavelength band. The peak located around 700 cm^−1^ corresponds to the stretching vibration mode of the B-O-B unit. The peak at 850–880 cm^−1^ is believed to be related to the connection of four non-bridging oxygen [SiO_4_] units. The peak located around 1150 cm^−1^ belongs to the stretching vibration of Si-O-Si bonds in the silicon oxygen tetrahedral unit. The peak in the 1250–1500 cm^−1^ region is related to the stretching vibration of the B-O bond in the [BO_3_] structural unit. The peak located around 1400 cm^−1^ corresponds to the [BO_3_] anti-symmetric stretching vibration mode. The absorption band at a wavenumber of 1640 cm^−1^ is caused by the bending vibration of H-O-H in free water. From Figure 5, it can be seen that as the amount of ZrB_2_ added increases, the number of [BO_3_] structural units in the heat-treated coating samples increases, indicating a higher formation of borosilicate glass.

**Figure 6:**
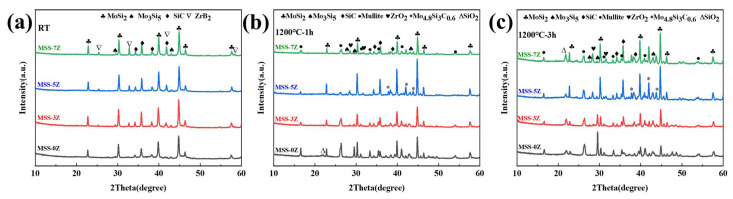
XRD patterns of single-layer coatings with different ZrB_2_ content before (**a**) and after calcination at 1200 °C for different periods of time: (**b**) 1 h; (**c**) 3 h.

The XRD patterns of single-layer coating with varying ZrB_2_ content at RT and after heat treatment at 1200 °C for 1 or 3 h in an air atmosphere are shown in Figure 6. In addition to the diffraction peaks of the three raw materials, a diffraction peak corresponding Mo_5_Si_3_, which is the oxidation product of MoSi_2_, is observed in the coating at RT (Figure 6a). The MoSi_2_ and SiC powders obtained through ball milling exhibit a remarkably reduced particle size, significantly increased specific surface area, and abundant defects, resulting in diminished oxidation resistance. The severe oxidation of SiC leads to a diminished peak intensity observed in the MSS-0Z sample spectrum, whereas MoO_3_, formed through the complete oxidation of MoSi_2_ and Mo_5_Si_3_, volatilizes and is absent from the XRD pattern (Figure 6b). The ZrB_2_ undergoes complete oxidation in the MoSi_2_–SiC–ZrB_2_ coatings after heat treatment, as evidenced by the detection of diffraction peaks corresponding to ZrO_2_ rather than ZrB_2_. The presence of the Mo_4.8_Si_3_C_0.6_ phase in the ZrB_2_-added coating indicates a reaction between SiC and Mo_5_Si_3_, facilitated by the flow of the borosilicate glass phase, which is recognized as the sole stable ternary phase within the Mo–Si–C system. The generation of Mo_4.8_Si_3_C_0.6_ is accompanied by the concurrent reduction of MoSi_2_, thereby maintaining a high peak strength for MoSi_2_ in MSS-5Z, while experiencing a decrease in the peak intensity of Mo_5_Si_3_ (Figure 6b). The Mo_4.8_Si_3_C_0.6_ crystal exists as a plethora of irregular lattices, which effectively enhance the asymmetry and lattice vibration of the structural unit. Moreover, owing to its favorable chemical compatibility with MoSi_2_ and SiC, Mo_4.8_Si_3_C_0.6_ can synergistically synergize with the glass phase to form a protective shell around the emitter particles, thereby effectively inhibiting further oxidation. As the content of ZrB_2_ increases, an excess B_2_O_3_ volatilizes, resulting in the formation of surface holes [49]. External heat flow can then intrude into the interior of the coating through these pathways and come into contact with the fiber, resulting in the increase in the mullite phase peak strength in MSS-7Z. After undergoing a 3 h heat treatment, an increased formation of Mo_5_Si_3_ was observed in the MSS-0Z sample, indicating enhanced oxidation of the emitter. Furthermore, the excessive presence of ZrO_2_ in the MSS-7Z coating can serve as nucleation sites within borosilicate glass, resulting in the crystallization of SiO_2_ [50]. The weak diffraction peak corresponding to SiO_2_ in the MSS-5Z spectrum suggests that both MoSi_2_ and SiC maintain their original states even after prolonged heating, thereby exhibiting excellent emissivity performance.

**Figure 7:**
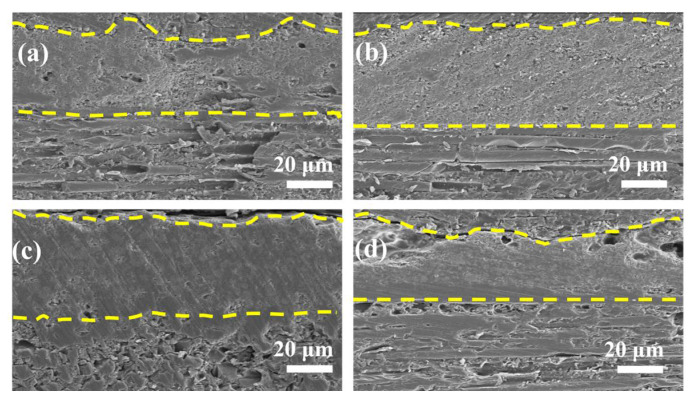
Cross-sectional SEM images of the fiber fabrics with the single-layer coatings after calcination at 1200 °C for 1 h: (**a**) MSS-0Z; (**b**) MSS-3Z; (**c**) MSS-5Z; (**d**) MSS-7Z.

The cross-sectional SEM images of the fiber fabrics with the single-layer coatings after calcination at 1200 °C for 1 h are shown in Figure 7. The two yellow dashed lines above and below in Figure 7 represent the interface between the coating and resin, as well as the interface between the coating and fiber cloth. The yellow dashed line in the middle is the coating, and the area above the coating is the fiber bundle. After sintering and particle rearrangement, obvious large and fine pores inside the MSS-0Z still remained, maintaining a conspicuous granular shape. The surface of the coating is somewhat concave, as the shrinkage of silica sol and the holes induce the collapse of the coating. The internal pores of MSS-3Z are reduced but still not dense, the MSS-5Z coating achieves densification from the viscous flow of the borosilicate glass phase, and the cross-section of the coating looks relatively smooth, with some molten glass liquid penetrating the interior of the fiber bundles. The portion of the MSS-7Z coating connected to the fiber is flat and compact, but large holes caused by the excessive volatilization of B_2_O_3_ still exist outside (Figure 7d). The thicknesses of the MSS-3Z and MSS-5Z coatings remained at approximately 50 µm after heat treatment, whereas the thicknesses of the other two decreased, which can be attributed to the volatilization of the oxidation products.

**Figure 8:**
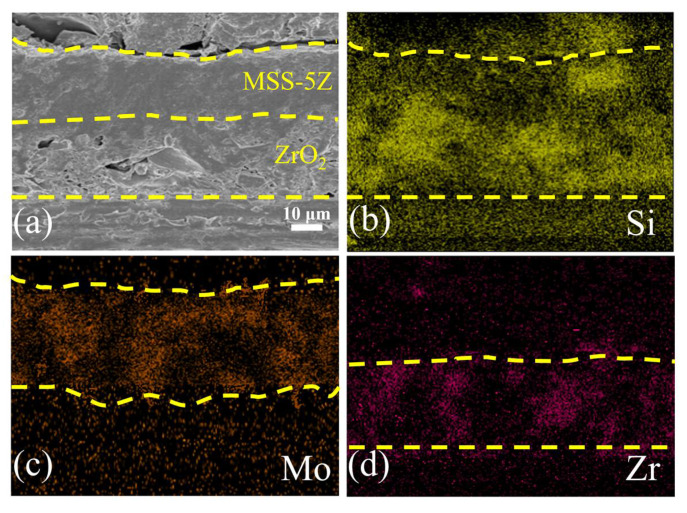
EDS elemental distribution images of a cross-section of the ASFF with a double-layer coating after calcination at 1200 °C for 1 h: (**a**) SEM image; (**b**) Si; (**c**) Mo; (**d**) Zr EDS spectra.

The cross-section and energy spectrum of the double-coated ASFF sample after heat treatment at 1200 °C for 1 h is shown in Figure 8. The range of the upper and lower layers of coating is roughly marked with yellow dashed lines in Figure 8. The components of the sample shown from bottom to top are the fiber bundles, ZrO_2_ coating, MSS-5Z coating, and resin curing of the sample for the cross-section shot. After heat treatment, the MSS-5Z coating is partially filled with borosilicate glass, which flows at high temperatures to make the outer layer dense without obvious voids. However, clear holes are still visible at the interface of the ZrO_2_ coating and fiber bundles. Due to the infiltration of the mobile glass phase, the holes at the interface of the ZrO_2_/MSS-5Z coating are also filled, making the interface indistinct. The layered interface between the coatings can be roughly distinguished by EDS elemental distribution (Figure 8c,d). Zr is mainly distributed in the ZrO_2_ layer, while the ZrO_2_ produced by the oxidation of ZrB_2_ is not clearly visible in the energy spectrum due to its low content.

### 3.3. Oxidation Reaction and Mechanism of Fillers in Coatings at High Temperatures

The primary cause for the deterioration of the coating performance lies in the oxidation of the emitter. The high-temperature stability of the MoSi_2_ and SiC emitters in the borosilicate matrix can be analyzed through a combination of reaction kinetics and thermodynamics. Based on the relevant literature and the XRD analysis, it is inferred that the following reactions will occur in the coating at high temperatures.

2/7MoSi_2_(s) + O_2_(g) = 2/7MoO_3_(s) + 4/7SiO_2_(s) (1)

5/7MoSi_2_(s) + O_2_(g) = 1/7Mo_5_Si_3_ (s) + SiO_2_(s) (2)

1/3MoSi_2_(s) + O_2_(g) = 1/3MoO_2_(s) + 2/3SiO_2_(s) (3)

1/2MoSi_2_(s) + O_2_(g) = 1/2Mo(s) + SiO_2_(s) (4)

2/5ZrB_2_(s) + O_2_(g) = 2/5ZrO_2_(s) + 2/5B2O_3_(s) (5)

1/2SiC(s) + O_2_(g) = 1/2SiO_2_(s) + 1/2CO_2_(g) (6)

SiC(s) + 12/7Mo_5_Si_3_(s) = 35/21Mo_4.8_Si_3_C_0.6_(s) + 4/7MoSi_2_(s) (7)

**Figure 9:**
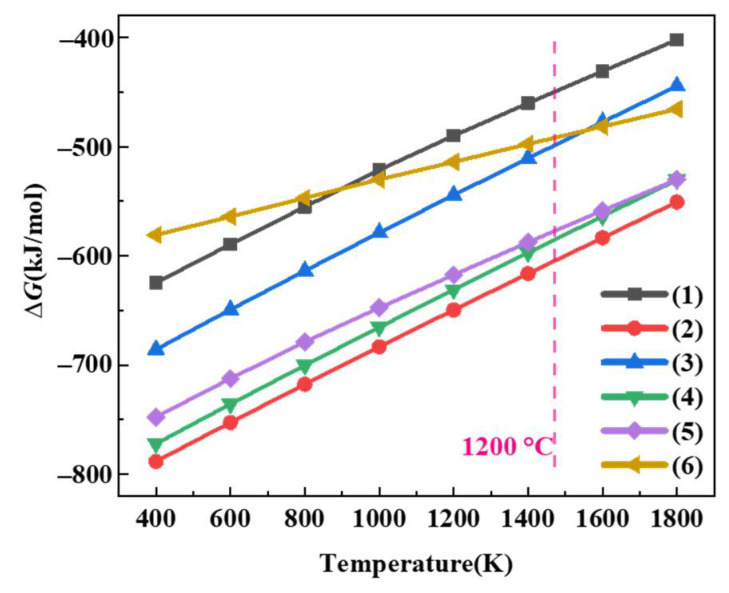
∆*G* of reactions (1)–(6) as a function of temperature.

The change in the Gibbs free energy (∆*G*) versus temperature for each reaction is calculated using the FACT program (Figure 9). It is unpractical to accurately calculate the ∆*G* of reaction (7) without Mo_4.8_Si_3_C_0.6_ data in the database. At 1200 °C, the ∆*G* of each reaction can be ordered as follows: reaction (1) > reaction (6) > reaction (3) > reaction (5) > reaction (4) > reaction (2). According to thermodynamic calculations, reaction (2), which generates Mo_5_Si_3_ as the by-product, has the lowest ∆*G* and is most likely to occur. Mo_3_Si_5_ was prone to further oxidation into MoO_3_, while MoO_3_ and MoO_2_ generated by reactions (1) and (3) volatilize at high temperature. Since the oxidation of MoSi_2_, SiC, and ZrB_2_ is a diffusion-controlled process, the reaction kinetics should also be considered. Existing research has confirmed that reaction (5) is susceptible to occur at temperatures above 1100 °C. Therefore, the oxidation of ZrB_2_ is completed in the initial stage of heating, and the generated B_2_O_3_ will form a eutectic mixture with SiO_2_ to create the borosilicate glass phase. In addition to oxidizing, part of the SiC will react with Mo_5_Si_3_ to generate Mo_4.8_Si_3_C_0.6_ and MoSi_2_.

**Figure 10:**
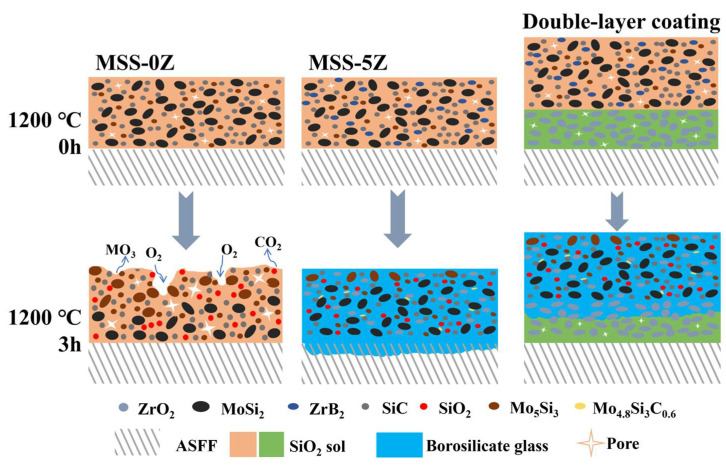
Oxidation mechanism diagram of the MSS-0Z, MSS-5Z, and double-layer coatings calcined at 1200 °C.

The oxidation mechanism diagrams of the MSS-0Z, MSS-5Z, and double-layer coatings calcined at 1200 °C are shown in Figure 10. Before heat treatment, the particles within each coating exhibit a strong interconnection facilitated by the adhesive properties of silica sol. However, numerous tiny pores are observed in the gaps between particle-to-particle stacking. Following a 3 h heat treatment, MoSi_2_ and SiC on the surface of MSS-0Z coating undergo oxidation to form Mo_5_Si_3_, MoO_3_, and SiO_2_ compounds. Nevertheless, due to inadequate softening at 1200 °C, SiO_2_ fails to effectively encapsulate the surfaces of emitter particles. The oxidation reaction, accompanied by significant volume expansion, the release of gaseous products, and the contraction of silica sol, includes extensive cracking within the coating. The gray arrows in Figure 10 represent the contact and release of gases such as O_2_ and CO_2_ with the coating surface. These cracks facilitate smooth infiltration and further promote the oxidation of internal SiC and MoSi_2_. In contrast, ZrB_2_ in the MSS-5Z coating exhibits preferential oxidation to form B_2_O_3_, effectively removing oxygen and generating a borosilicate glass phase with SiO_2_. Under high-temperature flow, the borosilicate glass phase exhibits a reduction in the diffusion rate of oxygen and effectively encapsulates the internal emitters (MoSi_2_ and SiC). The formation of oxidation products (i.e., Mo_5_Si_3_ and SiO_2_) is effectively reduced, while simultaneously preserving the morphological stability of the coating. Meanwhile, Mo_5_Si_3_ reacted with SiC to form the Mo_4.8_Si_3_C_0.6_ phase in the presence of the glass phase flow, instead of undergoing further oxidation to MoO_3_. The borosilicate glass phase formed in the double-coated sample at elevated temperatures facilitated the closure of voids within the coating under flow conditions, subsequently infiltrating the internal layer of the ZrO_2_ coating. Consequently, the two coatings were tightly bonded to facilitate interfacial melting and prevent glass-phase intrusion into the fiber bundles, thereby mitigating fiber degradation.

### 3.4. Analysis of Mechanical Properties of Single-Layer and Double-Layer Coatings after Heat Treatment at Different Temperatures

**Figure 11:**
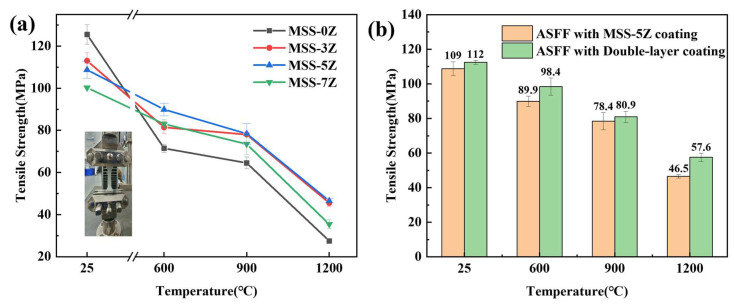
Tensile strength of the ASFF with coatings before and after calcination at different temperatures: (**a**) single-layer coatings; (**b**) MSS-5Z and double-layer coatings.

To investigate the influence of coatings with varying ZrB_2_ content on the tensile strength of the ASFF, the tensile strengths of the single-layer coated fiber fabrics were measured after calcination at different temperatures for 1 h (Figure 11a). With increasing heat treatment temperature, the mullite phase gradually precipitates, resulting in the formation of defects on the fiber surface and the coating undergoing localized sintering, which impedes the relative sliding between the fibers and produced axial stress. Thus, the overall tensile strength of the coated fiber fabrics decreases as the temperature increases. At RT, the tensile strength of the MSS-0Z coating is higher than that of the ASFF with the ZrB_2_ coating, reaching 125.5 ± 5 MPa. The decrease in silica sol content will affect the shaping of gel between fiber gaps, which in turn reduces the fracture of the gel layer and energy dissipates from the separation of the fiber–gel interface. Nonetheless, after high-temperature scorching, the mechanical properties of the ASFF with the MSS-0Z coating were relatively stable at 600–1200 °C, and the tensile strength decreased to about 27.5 MPa at 1200 °C. This is principally due to the oxidation of the emitter, which intensified with the increase in the calcination temperature. The oxidation of the emitter reduces the emissivity of the coating, which makes the ASFF surface gather more heat and accelerates the fiber crystallization. Generated SiO_2_ melts and penetrates the ASFF, and the subsequent brittle fracture of the ASFF leads to more mullite phase precipitation, defects, and damaged fibers. The tensile strength of the MSS-5Z-coated fabric after heat treatment is the highest among the samples and remains at about 46.5 MPa after burning at 1200 °C, which is 69% higher than that of the MSS-0Z-coated ASFF under the same conditions. ZrB_2_ mingled into the coating is oxidized to ZrO_2_, improving the toughness of the coating and the high-temperature oxidation resistance of the emitter. In addition, a high emissivity enhances the radiation of heat. Nevertheless, with the increase in ZrB_2_ content, the tensile strength of the MSS-7Z-coated fabric decreased sharply to about 35.4 ± 3 MPa after heat treatment at 1200 °C. Excess ZrB_2_ oxidizes the borosilicate glass phase penetrating between the fiber bundles and excessive ZrO_2_ as the nucleation site causes glass crystallization, which is not conducive to the mutual removal of fibers.

The tensile strength of the double-layer coated ASFFs with an intermediate layer added on the foundation of the single-layer MSS-5Z coating were measured at different temperatures after insulation for 1 h and then were further compared (Figure 11b). The double-layer coated ASFF exhibited consistently higher tensile strength across all measured temperatures. Considering relevant studies, we believe that the transformation from t-ZrO_2_ to m-ZrO_2_ occurred under tensile strain. During the phase transition, energy is absorbed, stress at the crack tip diminishes, and crack propagation is inhibited. The tensile strength of the fabric increased by 11.1 MPa (23.9%) from 46.5 to 57.6 MPa at 1200 °C. The intermediate ZrO_2_ coating, through phase transition toughening, effectively prevents infiltration of molten borosilicate glass between fiber bundles, thereby reducing fiber damage. Additionally, the thickness of the coating has an impact on the fabric’s tensile strength.

**Figure 12:**
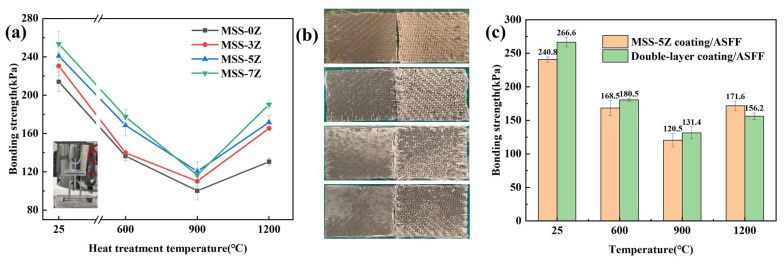
(**a**) Bonding strengths of the single-layer coating/ASFF. (**b**) Photo of samples after pull-out tests. (**c**) Bonding strengths of MSS-5Z coating/ASFF and double-layer coating/ASFF.

The bonding strength between the ASFF and the coating with different ZrB_2_ contents after calcination at different temperatures for 1 h is shown in Figure 12a. The bonding strength first decreases and then increases roughly with the heat treatment temperature. The oxidation of SiC and MoSi_2_ after heat treatment at 600 °C or 900 °C is accompanied by the generation of gaseous products and significant volume expansion that allows for voids and defects to appear at the ASFF/coating interface that are not close in proximity. The bonding strength of the ASFF with MSS-0Z coating is 100.2 ± 9 kPa at 900 °C. Silica sol functions as an adhesive to provide robust cohesion at 1200 °C, and the bonding strength increases to 130.5 ± 4kPa. As the ZrB_2_ content increased, a more fused borosilicate glass phase was generated to further enhance the bonding ability. Moreover, the higher proportion of B/Si in borosilicate glass led to a decrease in viscosity and improved flowability. At 1200 °C, the bonding strength of ASFF/MSS-7Z reached 190.3 kPa, which was 45.8% higher than that of the MSS-0Z sample. Sample photographs of the coated ASFFs after heat treatment at 1200 °C for 1 h following the bond strength test are shown in Figure 12b. The strong bonding effect of borosilicate glass is clearly observed, while the coating is more inaccessibly completely separated from the ASFF after testing with increasing ZrB_2_ content.

The bonding strengths of the MSS-5Z and double-layer coatings after calcination for 1 h and pull-out tests are shown in Figure 12c. The bond strength of double-layer coating/ASFF below 900 °C is higher than that of the single-layer coating/ASFF, which is 10.7%, 7.1%, and 9.1% higher at RT, 600 °C, and 900 °C, respectively. The contact part is the ZrO_2_ layer, which will not cause the coating to peel off the oxidative expansion of the MoSi_2_ powder at elevated temperatures. The borosilicate glass phase produced by the upper layer penetrates downward into the ZrO_2_ layer and will not reinforce the interfacial connection between the fiber bundle and the coating. Hence, the binding strength of the double-layer coating/ASFF (156.2 ± 5 kPa) is lower than that of the MSS-5Z sample.

### 3.5. Comparison of Thermal Insulation Performance between Single-Layer and Double-Layer Coatings

**Figure 13:**
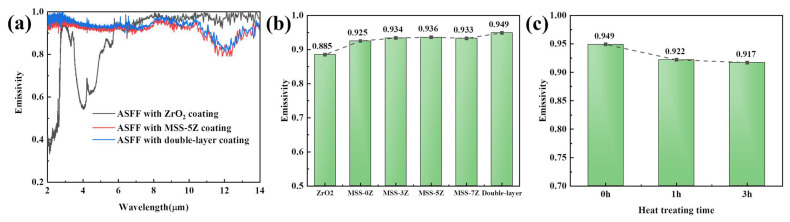
(**a**) Emissivity of the raw ASFF with a ZrO_2_ coating, ASFF with an MSS-5Z coating, and ASFF with a double-layer coating. (**b**) Average spectral emissivity of the coated ASFF in the wavelength range of 2~14 μm at RT. (**c**) Emissivity of the ASFF with a double-layer coating after heat treatment at 1200 °C for different times.

Figure 13a shows the spectral emissivity of the ASFF sprayed with the ZrO_2_, MSS-5Z, and double-layer coatings at RT in the wavelength range of 2–14 μm. The spectral emissivity of the MSS-5Z and the double-layer coatings show similar trends with wavelength, but an obvious distinction is observed with the ZrO_2_ coating. The ZrO_2_ coating exhibits a lower average emissivity in the range of 2–6 μm due to several strong reflection peaks, whereas MSS-5Z and double-layer coated ASFFs have an emissivity above 0.92 in this range, meeting the requirement for radiant TPS. In the 7–14 μm band, the emissivity trend is exactly the opposite, and the ZrO_2_ coating has a higher emissivity. According to Wien’s displacement law, as the temperature increases, blackbody radiation is principally concentrated in the short-wavelength range. The high-emissivity coating should significantly advance the emissivity in the low band, which is of immense significance for its service at high temperatures. In the emissivity tests, the absorption or reflection of electromagnetic waves by the external coating is prioritized before penetrating the interior of the coating. Therefore, the test mainly collects the reflection effect of the surface coating on electromagnetic waves, and the collaborative absorption effect of the internal coating has less impact. In the 10–14 μm wavelength range, the emissivity curve of MSS-5Z is groove-shaped, and the emissivity decreases by approximately 0.1 due to the inherent defects of SiC particles [51]. Figure 13b shows the average spectral emissivity of different samples without heat treatment at RT. The average emissivity of the ZrO_2_-coated ASFF is only 0.885. The average emissivity of coatings containing the emissivity filler (i.e., MoSi_2_ and SiC) is above 0.925, among which the average emissivity of the double-layer coating is the highest (0.949), indicating that the emissivity is affected by several factors simultaneously, such as chemical composition and coating thickness.

The average spectral emissivity of the double-coated ASFF treated at 1200 °C for different times is shown in Figure 13c. The emissivity of the coating showed a decreasing trend with the increase in the heat treatment time. When the heat treatment time reaches 3 h, the surface emissivity of the ASFF remains above 0.91, demonstrating its high emissivity property. The surface of the coating with ZrB_2_ added forms a silicon-based glass layer at high temperatures. This amorphous structure of long-range disorder can form localized states, whose energy provides conditions for electron transition in the bandgap and promotes short-wave infrared absorption [52]. In addition, the glass layer can mend the pores on the surface of the coating, preclude the entry of oxygen, and improve the oxidation resistance of the MoSi_2_ and SiC emitters.

**Figure 14:**
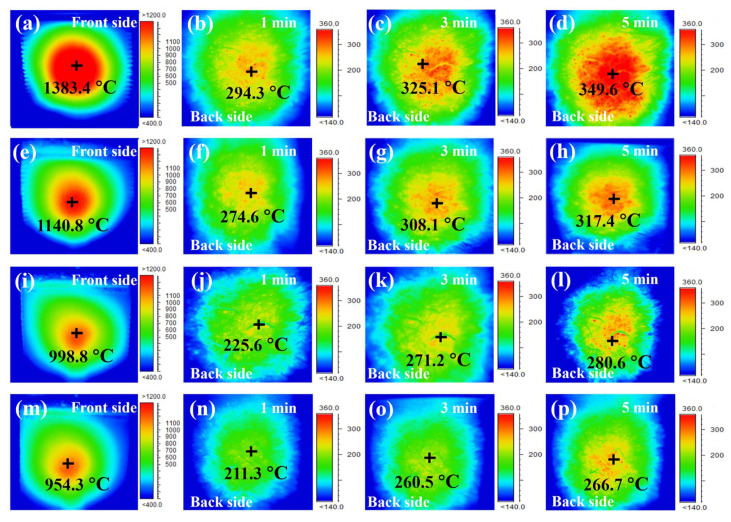
Infrared thermal image of flexible insulation felt: (**a**–**d**) without coating; (**e**–**h**) MSS-0Z coating; (**i**–**l**) MSS-5Z coating; (**m**–**p**) double-layer coating.

The thermal insulation ability of the raw and coated fiber fabrics was characterized using an infrared thermal camera to record thermograms of the surface and back of the sample (Figure 14). A layer of ASFF was covered on the cotton fiber core, the size of the fiber cloth was 100 × 80 mm, and the thickness was 10 mm. The flexible felt fiber was placed 120 mm in front of the butane flame spray gun and heated for 5 min until the temperature of the backside had stabilized. After burning the original ASFF under a flame for 1 min, the maximum temperature at the front center of the ASFF remained stable at around 1383.4 °C, while the back maximum temperature was approximately 294.3 °C (Figure 14a,b). When the ignition time reached 5 min, the back maximum temperature remained stable at about 349.6 °C (Figure 14d). When the MSS-0Z coating was sprayed on the ASFF, the front and back temperatures stabilized at about 1140.8 and 317.4 °C after 1 and 5 min of scorching, respectively (Figure 14e,h). The surface temperature of coated ASFF during the heating process is much lower than that without the coating. This is because the high-emissivity coating can disperse part of the heat on the surface. After 5 min of ignition, the front temperature of the MSS-5Z-coated ASFF decreased by 27% compared to the original ASFF, stabilizing at about 998.8 °C (Figure 14i), while the backside temperature stabilized at 280.5 °C (Figure 14l), decreasing by about 19.8%. The borosilicate glass phase can fill the holes, effectively preventing the further oxidation of the emitter. For high-temperature thermal protection composite materials, the radiation heat transfer mechanism plays a more dominant role than heat conduction and heat convection. A high emissivity corresponds to more heat being radiated out at the same temperature, which reduces the surface temperature of the ASFF. The thickness of the coating also affected the thermal insulation performance, with the thicker double-layer coating providing lower maximum front (954.3 °C) and back (266.7 °C) temperatures under a stable butane flame (Figure 14m,p). According to Figure 13b, a higher emissivity could radiate more surface heat, and a thicker coating reduced the efficiency of heat transfer to the back of the insulation felt. Multiple factors collectively determined that double-layer coatings had better thermal insulation performance.

## 4. Conclusions

In this study, a glass phase that can fill the internal voids of an ASFF coating was formed by the modification of ZrB_2_ at high temperatures to leverage the synergistic effect of high-emissivity MoSi_2_ and SiC fillers and promote the oxidation resistance of the emitter. The addition of a ZrO_2_ intermediate layer between the ASFF and the high-emissivity coating effectively raised the tensile strength of the fiber fabric. Compared with the single-layer MSS-5Z-coated ASFF, the tensile strength of the double-coated fabric is 23.9% higher and its binding strength reached 156.2 kPa. The ZrO_2_ sol layer can not only absorb the stress in the tensile strain caused by the phase transition toughening mechanism but also prevent the glass phase from penetrating the fiber bundle and is instrumental in interface debonding and fiber pull-out. Moreover, the average spectral emissivity of the double-coated ASFF after heat treatment at 1200 °C for 3 h is still above 0.91, which can meet the high-temperature thermal insulation performance demands of TPS. After butane flame heating for 5 min, the backside temperature of the double-layer coating felt fiber was 83 °C (23%) lower compared with the bare ASFF. The structural design of the ZrB_2_-doped coating and intermediate layer not only reduces the oxidation rate but also blocks the bonding between the outer coating and fiber bundles, which effectively restrains the strength attenuation of the high-emissivity coating after high-temperature application. Further exploration can be conducted on the introduction of reflective planes in coatings or felt fiber to reduce the diffusion of thermal energy into the interior of the aircraft, which is also an important mechanism of TPS.

## Figures and Tables

**Table 1:** Compositions of the MoSi_2_–SiC–ZrB_2_ coating and ZrO_2_ coating.

Sample	Composition (wt.%)				
	MoSi_2_	SiC	ZrB_2_	ZrO_2_	SiO_2_-Sol
MSS-0Z	25	25	0	0	50
MSS-3Z	25	25	3	0	47
MSS-5Z	25	25	5	0	45
MSS-7Z	25	25	7	0	43
ZrO_2_ coating	0	0	0	50	50

## Data Availability

Data are contained within the article.
